# Supplementary material for: Assessment of knowledge, attitudes, and practices among community pharmacists in Lahore regarding antibiotic dispensing without prescription: A cross-sectional study
Source: PLoS One. 2024 Jun 13;19(6):e0304361. doi: 10.1371/journal.pone.0304361 (PMC11175427; doi:10.1371/journal.pone.0304361)
Supplement: Attachment — Submitted filename: Comments File for DAwP.docx [file pone.0304361.s001.docx]

Comment 1: In the Methodology Section, at line 133, the abbreviations "CPs" and "DAwP" are utilized. I would recommend expanding these abbreviations to enhance reader comprehension.

Comment 2: Within the Methodology Section, it would be beneficial to provide further clarification regarding the sample size. Specifically, it would be helpful to explain how the sample size was determined and indicate the source of the calculation.

Comment 3: In the Questionnaire Development Section, at Line 152, additional clarification is needed regarding the version of SPSS software used. The abstract mentions the use of SPSS version 26 for statistical analysis, whereas here, it is stated as the 21st version. This discrepancy should be addressed.

Comment 4: Throughout the entire manuscript, I recommend a thorough review to identify and rectify any grammatical mistakes, spelling errors, and typographical errors.

Comment 5: Additionally, please check the formatting of the manuscript to ensure it adheres to standard guidelines. Remove any extraneous lines or spacing issues, such as those found at lines 256, 257, 377, and 529, to maintain a professional appearance.
