# Supplementary material for: Assessment of knowledge, attitudes, and practices among community pharmacists in Lahore regarding antibiotic dispensing without prescription: A cross-sectional study
Source: PLoS One. 2024 Jun 13;19(6):e0304361. doi: 10.1371/journal.pone.0304361 (PMC11175427; doi:10.1371/journal.pone.0304361)
Supplement: Attachment — Submitted filename: Response to Reviewers.docx [file pone.0304361.s002.docx]

**Rebuttal Letter PlosOne Reviewer Comments** 'Response to Reviewers'

**Reviewer 1:**

***Comment 1: In the Methodology Section, at line 133, the abbreviations "CPs" and "DAwP" are utilized. I would recommend expanding these abbreviations to enhance reader comprehension.***

**Answer:** Thank you for your suggestion. We have revised the manuscript to comprehensively spell out the abbreviations "CPs" and "DAwP" at their initial use in the Methodology Section, line 133, to improve clarity and understanding for our readers.

***Comment 2: Within the Methodology Section, it would be beneficial to provide further clarification regarding the sample size. Specifically, it would be helpful to explain how the sample size was determined and indicate the source of the calculation.***

**Answer:** Thank you for highlighting this aspect. We have revised the Methodology Section to include a more detailed and clear explanation of how the sample size was determined and the source of the calculation to ensure a comprehensive understanding.

***Comment 3: In the Questionnaire Development Section, at Line 152, additional clarification is needed regarding the version of SPSS software used. The abstract mentions using SPSS version 26 for statistical analysis, whereas it is the 21st version here. This discrepancy should be addressed.***

**Answer:** Thank you for drawing attention to this inconsistency. The discrepancy in the SPSS software version mentioned arose because one author used version 26 while the other had version 21. We have now updated the manuscript to consistently refer to SPSS version 26, as this was the version used for the final revision and analysis of the data. We appreciate your keen observation in this matter.

***Comment 4: Throughout the entire manuscript, I recommend a thorough review to identify and rectify any grammatical mistakes, spelling errors, and typographical errors.***

**Answer:** Thank you for emphasizing the importance of accuracy in our manuscript. We appreciate your attention to detail and have comprehensively reviewed the document to correct grammatical, spelling, and typographical errors. Your feedback has been instrumental in enhancing the quality of our work.

***Comment 5: Please check the manuscript's formatting to ensure it adheres to standard guidelines. Remove extraneous lines or spacing issues, such as those found at lines 256, 257, 377, and 529, to maintain a professional appearance.***

**Answer:** Thank you for pointing out the formatting inconsistencies in our manuscript. We have thoroughly reviewed and corrected the formatting, explicitly addressing the issues at lines 256, 257, 377, and 529, to ensure that our document meets the standard guidelines and is presented professionally. Your input has been invaluable in refining the manuscript.

**Reviewer 2:**

***Title:
The title of the manuscript needs revision as the terms "Prescription precision" and "antibiotic adherence" do not align with the study's objectives.***

**Answer:** Thank you for your attention to the title of our study. We are grateful for your input and have revised the title to "***Assessment of Knowledge, Attitudes, and Practices among Community Pharmacists in Lahore Regarding Antibiotic Dispensing without Prescription: A Cross-Sectional Study."***

***Background:***

***It is suggested to use antibiotic resistance instead of "antimicrobial resistance" when especially denoting resistance from bacterial infections. The terminology should be coherent throughout.***

**Answer:** Thank you for your recommendation regarding the use of terminology. We have updated our manuscript to consistently use "antibiotic resistance" in the context of bacterial infections, ensuring coherence throughout the document. Your input is greatly appreciated.

***Please provide more details on how irrational antibiotic practices have contributed to antibiotic resistance in Pakistan and what innovative findings your study presents that add to the existing literature.***

**Answer:** Thank you for your inquiry about the impact of irrational antibiotic practices on resistance in Pakistan. Our study focuses specifically on the community level, where we observed a concerning trend: restricted and high-potency antibiotics are frequently dispensed without prescriptions. This indiscriminate dispensing practice is a significant factor in the irrational use of antibiotics, contributing to the rise of antibiotic resistance. Our findings shed light on this critical issue, adding a new dimension to the existing literature by highlighting the need for more stringent controls and awareness at the community pharmacy level.

**Introduction:**

***The references in the introduction section need updation—for instance, references # 2,3,4,5 and 6. To provide a more holistic background to antibiotic resistance in Pakistan and the factors contributing to it, the following study may provide a more solid rationale for the objectives of your research. (This is optional and should be taken as a suggestion for the improvement of the manuscript)***

***PMCID: PMC83887772. PMCID: PMC7602895***

**Answer:** Thank you for your suggestion. We've updated our manuscript with one of the recommended PMCIDs to reflect antibiotic resistance better in Pakistan. Your input has been invaluable in enhancing our study.

***Methodology:***
***The manuscript provides no details on the total population size of CPs used and how the sample size is calculated. Are medical stores included in the calculation of sample size? Hence, mention the inclusion/exclusion criteria in detail.***

**Answer:** Thank you for highlighting the need for clarity regarding our manuscript's population size and sample size calculation. We have now included detailed information on these aspects, along with explicit inclusion and exclusion criteria, which also address the role of medical stores in our study. This addition ensures a more comprehensive understanding of our research methodology.

***Also, provide more clarity on the development and validity of your questionnaire. How many items (added/deleted/modified) are from the idea generation phase?***

**Answer:** Thank you for your feedback. We've addressed your concerns in our manuscript, specifically detailing our questionnaire's development and validation process. Initially, the questionnaire comprised 33 items, which, after careful consideration and pre-testing, were refined to 27. Furthermore, we have applied Cronbach's alpha to ensure the validity of the questionnaire, and this information has been duly added for clarity.

***The figures provided on the total community pharmacies in Pakistan are taken from a 2017 study. Have the statistics remained the same until now? If not, would they affect the calculation of sample size? Please justify.***

**Answer:** Thank you for pointing out the dated figures. Our study targets 3618 community pharmacies in Lahore, with a margin of ±10, which exceeds our required sample size. This ensures our sample size calculation remains valid despite potential changes in the total number of pharmacies since 2017.

***For lines 171-173: For a better understanding, please provide details on how frequent visits were made to achieve the desired response rate.***

***Due to the high frequencies of the populations and a low number of pharmacies, the dispensing will not be appropriate, and wrong dispensing and without prescription dispensing are more frequent.***

**Answer: Thank you for your feedback on lines 171-173. To enhance clarity, we have now included specific details in our manuscript regarding the frequency of visits conducted to achieve the desired response rate. This addition addresses the concerns about the impact of high patient populations and limited pharmacy numbers on dispensing practices, ensuring a more comprehensive understanding of the study's context.**

**Discussion:**

***In the discussion section, when we discuss antibiotic resistance, it is crucial to discuss antimicrobial stewardship. However, no such mention can be seen.***

***The references used throughout the discussion section need updation. For instance, in ref 28 and 52, when discussing how undergraduate training may affect the practices of CPs on antibiotic dispensing, the following updated study can be a relevant and appropriate reference to your study. (This is optional and should be taken as a suggestion for the improvement of the manuscript)
PMCID: PMC8532898***

**Answer:** Thank you for your feedback. We have now integrated discussions on antimicrobial stewardship into our analysis of antibiotic resistance in the revised discussion section. Additionally, references, particularly ref 28 and 52, have been updated with the suggested study (PMCID: PMC8532898) to enrich our examination of CPs' practices influenced by undergraduate training. Your suggestions have significantly enhanced the manuscript's comprehensiveness and relevance.
