# Supplementary material for: Assessment of knowledge, attitudes, and practices among community pharmacists in Lahore regarding antibiotic dispensing without prescription: A cross-sectional study
Source: PLoS One. 2024 Jun 13;19(6):e0304361. doi: 10.1371/journal.pone.0304361 (PMC11175427; doi:10.1371/journal.pone.0304361)
Supplement: Attachment — Submitted filename: Comments for the authors-PLOS ONE.docx [file pone.0304361.s003.docx]

**Comments for the authors**

**Assessment of Knowledge, Attitudes, and Practices among Community Pharmacists in**

**Lahore Regarding Antibiotic Dispensing without Prescription: A Cross-Sectional Study**

**Introduction**

**Comment 1**

**Page 6, line 129-130:** Consider providing more specific details regarding the aim of the present study that will give a distinction from the previous publications within the same area and scope.

**Discussion**

**Comment 2**

**Page 13, line 250: ‘’…**were significantly 43.2% less likely…’’

Consider mentioning the times rather than the percentage.

**Comment 3**

**Page 17, line 315-316: ‘’** The present study further revealed that the CPs had good practices towards DAwP. Most CPs never or sometimes warn the patients about the potential side effects of medicines when dispensed with antibiotics without a prescription.’’

Consider revising and rephrasing these sentences as there is an obvious conflict between the good practices and never or sometimes providing warning to the patients about the potential side effects!

**Comment 4**

**Page 17, line 323: ‘’** This might be because the pharmacy profession is in its infancy in Pakistan.’’

What are the authors trying to say in this sentence. Is it correct that the pharmacy profession is in infancy time in this country!

**Strength and limitations**

**Comment 5**

**Page 19, line 351: ‘’** no previously published data is available in any South Asian’’

Authors are required not to generalize their findings to the surrounding geographical area as the main scope of the study was related to Pakistan.

Consider removing this sentence.

**Comment 6**

**Page 19, line 355: ‘’**but also worldwide’’.

Similarly, authors are required not to generalize their findings as the main scope of the study was related to Pakistan. Consider removing this sentence.

**Conclusion**

**Comment 7**

**Page 17, line 365: ‘’**The present study concluded that CPs had good knowledge and positive attitudes towards DAwP’.

Consider rephrasing this sentence. Since, based on the current study findings, there is a relatively good knowledge but poor attitudes towards DAwP’’. Consider mentioning this point as a part of the conclusion within the conclusion of both the abstract and main text.

**References**

**Comment 8: Consider revising and correcting the following list of references.**

Reference (2): This reference is outdated. Consider providing a more recent one.

Reference (3): Provide the website link.

Reference (6): This reference is outdated. Consider providing a more recent one.

Reference (8): Provide the website link.

Reference (9): This reference is outdated. Consider providing a more recent one, including the one

doi: 10.3855/jidc.13066.

Reference (10): This reference is unrelated to the sentence reported. Consider correction.

Reference (23): Provide the website link.

Reference (28): Consider removing the data from this reference and replacing a more appropriate recent one with data within its reference.

Reference (30): Provide the website link.

Reference (36): This reference is unrelated to the sentence reported. Consider correction.
