# Supplementary material for: Assessment of knowledge, attitudes, and practices among community pharmacists in Lahore regarding antibiotic dispensing without prescription: A cross-sectional study
Source: PLoS One. 2024 Jun 13;19(6):e0304361. doi: 10.1371/journal.pone.0304361 (PMC11175427; doi:10.1371/journal.pone.0304361)
Supplement: Attachment — Submitted filename: Comments for the authors.docx [file pone.0304361.s005.docx]

**Assessment of Knowledge, Attitudes, and Practices among Community Pharmacists in**

**Lahore Regarding Antibiotic Dispensing without Prescription: A Cross-Sectional Study**

**Comments for the authors**

There are still remaining critics that did not get into consideration and corrected by the authors, as below:

**Introduction:**

Page 5: line 15-17: *“Studies depict that such irrational dispensing practices are also commonly*

*found in various Gulf countries like Saudi Arabia (14, 15), Egypt (16), Jordan (17), and Syria*

*(18)”.*

The sentence is incorrect and needs to be observed and rephrased since some of the countries are not a part of the Gulf, such as Egypt, Jordan and Syria.

The authors can mention Gulf countries like Saudi Arabia with the citations (14, 15). Also it is better to consider another example of the Gulf countries and Middle East like Iraq with the citation (doi: 10.3855/jidc.13066). Then it can mention other countries, like Egypt, Jordan and Syria.

**References**

Reference (3): Invalid link. Provide the correct website link.

Reference (30): Invalid link. Provide the correct website link.

Reference (28): Incorrect. Consider corrected one.
