# Supplementary material for: Assessment of knowledge, attitudes, and practices among community pharmacists in Lahore regarding antibiotic dispensing without prescription: A cross-sectional study
Source: PLoS One. 2024 Jun 13;19(6):e0304361. doi: 10.1371/journal.pone.0304361 (PMC11175427; doi:10.1371/journal.pone.0304361)
Supplement: Attachment — Submitted filename: Response to Reviewers.docx [file pone.0304361.s006.docx]

**Rebuttal Letter PlosOne Reviewer Comments** 'Response to Reviewers'

**Assessment of Knowledge, Attitudes, and Practices among Community Pharmacists in Lahore Regarding Antibiotic Dispensing without Prescription: A Cross-Sectional Study**

**Introduction**

***Comment 1***
***Page 5: line 15-17:*** *“Studies depict that such irrational dispensing practices are also commonly found in various Gulf countries like Saudi Arabia (14, 15), Egypt (16), Jordan (17), and Syria (18)”.*

*The sentence is incorrect and needs to be observed and rephrased since some of the countries are not a part of the Gulf, such as Egypt, Jordan and Syria.*

*The authors can mention Gulf countries like Saudi Arabia with the citations (14, 15). Also it is better to consider another example of the Gulf countries and Middle East like Iraq with the citation (DOI: 10.3855/jidc.13066). Then it can mention other countries, like Egypt, Jordan and Syria.*

**Answer:** Thank you for considering this point. I have made revisions to the sentences to ensure they are clear and easily understood.

**References**

***Comment 2: Consider revising and correcting the following list of references.***

**Answer:** Thank you for addressing the issues. I have rectified all concerns related to the references.

Reference (3): Provided the correct website link.

Reference (28): Provided the correct website link.

Reference (30): Provided the correct website link.
